# Effect of acupuncture at 3 anti-fatigue acupoints in the treatment of cancer-related fatigue in patients with cancer: Protocol for a systematic review and meta-analysis of randomized controlled trials

**DOI:** 10.1097/MD.0000000000015919

**Published:** 2019-06-07

**Authors:** Muxi Liao, Yizi Xie, Jiao Yan, Tong Lin, Shuliang Ji, Zongyao Li, Wenjing Zhao, Yaqin Yang, Lizhu Lin, Jietao Lin

**Affiliations:** a The First Affiliated Hospital of Guangzhou University of Chinese Medicine; b First Clinical College, Guangzhou University of Chinese Medicine, Guangzhou; c Shenzhen Pingle Orthopaedic Hospital, Shenzhen; d Oncology Center, The First Affiliated Hospital of Guangzhou University of Chinese Medicine, Guangzhou, China.

**Keywords:** acupuncture, cancer-related fatigue, meta-analysis, protocol, systematic review

## Abstract

**Background:**

Cancer-related fatigue (CRF), is a common distressing symptom of cancer. What's more, “Three anti-fatigue acupoints” is one of the most important components of “Jin's 3-needle therapy” created by Rui Jin, a professor of Guangzhou University of Chinese Medicine, which can be used in the treatment of CRF. In this article, researchers will assess the safety and effect of acupuncture at 3 anti-fatigue acupoints on CRF in patients with cancer.

**Methods:**

Literature search for relevant articles up to October 2018 will be carried out in 9 databases: Cochrane Library, Embase, PubMed, VIP, CBM, CNKI, Wanfang Database, CiNii, and OASIS. The included literatures will be randomized controlled trials of acupuncture at 3 anti-fatigue acupoints on CRF in patients with cancer. The certain common scales, which reflect the patients’ fatigue degree or life quality will be the primary outcome measures. The secondary outcome measures will be defined with the blood index. After collecting the data, we will utilize Stata V.13.0. to perform data synthesis, subgroup analysis, partial sequence analysis, sensitivity analysis, and so on. A funnel plot will be used to assess reporting biases. And the funnel plot will be evaluated by the Egger and Begg tests. The quality of evidence will be judged by the grading of recommendations assessment, development, and evaluation.

**Results:**

The results of this systematic review and meta-analysis will be published in a peer-reviewed journal.

**Conclusion:**

Our study will provide the evidence for the clinical efficacy and safety of acupuncture at 3 anti-fatigue acupoints in the treatment of CRF.

**Key Points**

- This study will assess the clinical efficacy and safety of acupuncture at 3 anti-fatigue acupoints in the treatment of cancer-related fatigue.
- Two reviewers will independently conduct the data extraction and risk f bias assessment.
- The grading of recommendations assessment, development, and evaluation system will be applied to further evaluate study findings.
- There may be a language bias, as both English and Chinese studies will be included.
- There may be clinical heterogeneity due to variations in treatment frequency and duration and the use of additional therapies.

## 1 Introduction

### 1.1 Description of the condition

Cancer-related fatigue (CRF), is a common distressing symptom of cancer. Over 99% of patients experienced some levels of fatigue during treatment with radiation, chemotherapy, and biologic therapies.^[1]^ Although fatigue is the most prevalent in patients undergoing chemotherapy or those with advanced diseases, it is also an important problem among cancer survivors.^[2,3]^ Fatigue mostly resolves in the year after treatment completion, but approximately 30% of patients experience more persistent fatigue that may last for 10 years or more.^[4]^ Studies have shown that CRF affects multiple aspects of individuals’ lives, such as performing activities of daily living, decreasing functional status, and limiting overall quality of life.^[5,6]^ In addition, CRF is associated with decreased survival and interferes with employment, family life, social relationships as well as sexual life.^[7–9]^ One of the consistent and key symptoms of CRF is seemingly the presence of emotional distress or mood disturbance.^[10,11]^ It has been reported that erythropoietin can reduce the need for transfusions, decrease fatigue levels, and improve quality of life in patients receiving chemotherapy.^[12–16]^ However, recent evidence has raised concerns regarding the safety of erythropoietin, suggesting it should not be used for the management of CRF.^[17]^ Fortunately, traditional Chinese medicine (TCM), such as acupuncture, has been gradually acknowledged worldwide for the management of CRF.^[18]^

### 1.2 Description of the intervention

**Figure 1:**
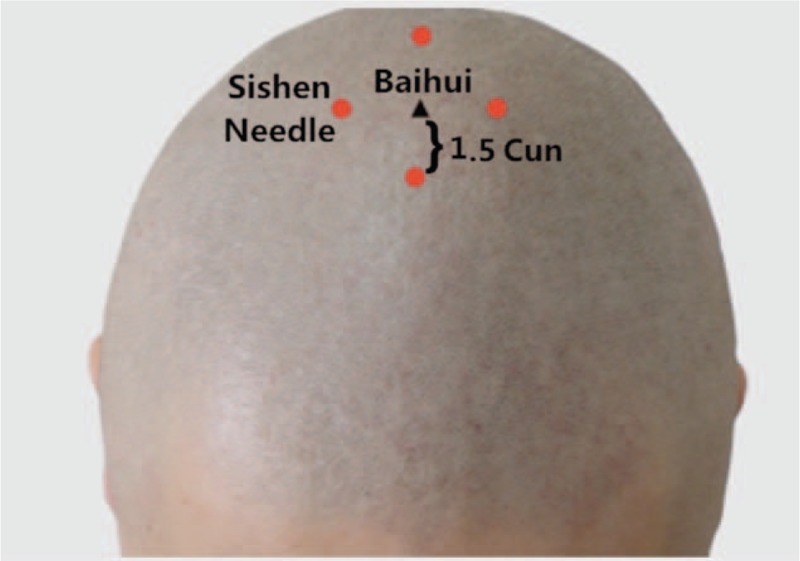
The location of Sishen Needle.

**Figure 2:**
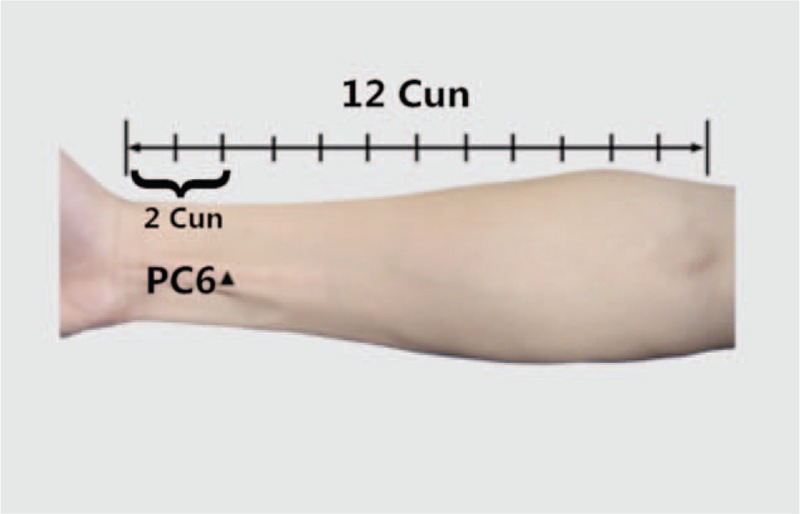
The location of PC6.

**Figure 3:**
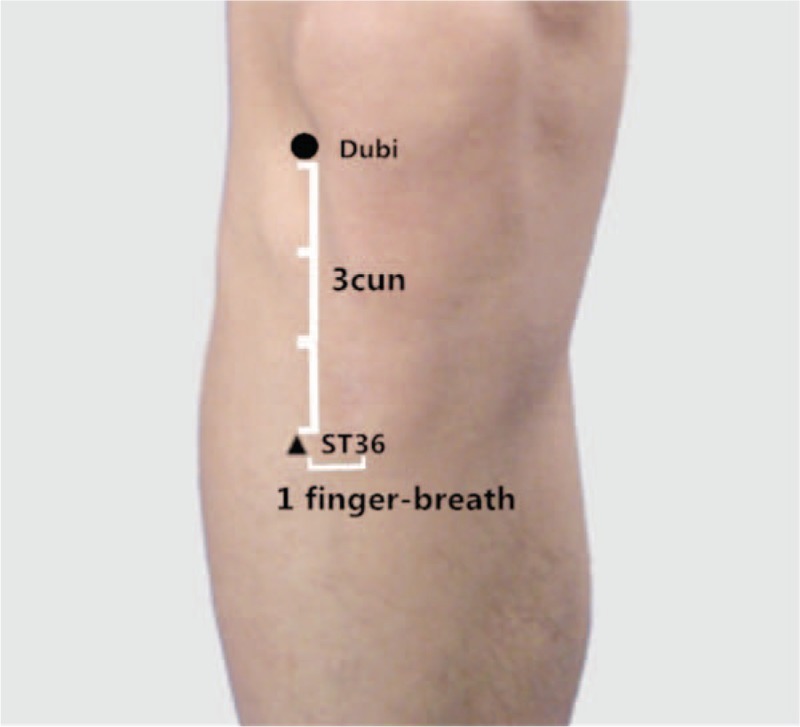
The location of ST36.

Acupuncture, as an indispensable part of TCM, has been proved to be effective for CRF management and should be recommended as a beneficial alternative therapy for CRF patients. The main therapeutic effects of acupuncture include dredging the meridians (channels and collaterals), regulating Yin and Yang, and strengthening the body's resistance to eliminate pathogenic factors.^[19]^ Three anti-fatigue acupoints is an acupoint set consisting of Sishen Needle, Neiguan (PC6), Zusanli (ST36).^[20]^ Sishen Needle is located on the parietal region, 1.5 cun anterior, posterior and lateral to Baihui, 4 acpoints totally, which is shown in Figure 1. PC6 is located 2 cun above the transverse crease of the wrist, between the tendons of palmaris longus and flexor carpi radialis. ST36 is located 3 cun directly below Dubi, and 1 finger-breath lateral to the anterior border of the tibia. Cun is defined based on the rules of traditional acupuncture as the width of the interphalangeal joint of the patient's thumb.^[21]^ The location of PC6 and ST36 is shown in Figures 2 and 3. Several clinical trials have shown that PC6 or ST36 is effective in treating CRF.^[22]^ However, the effect of acupuncture at 3 anti-fatigue acupoints in the treatment of CRF has not been investigated. In addition, there is insufficient evidence to support the widespread use of acupuncture at 3 anti-fatigue acupoints. Consequently, an examination of this therapy's effect on CRF is needed. Therefore, we aim to carry out a systematic review and meta-analysis to evaluate current evidence on the effects of acupuncture at 3 anti-fatigue acupoints in the treatment of CRF in patients with cancer. Researchers will include randomized controlled trials (RCTs) with no language restrictions that compare the effects of acupuncture or electroacupuncture with usual care or placebo acupuncture.

## 2 Methods

### 2.1 Selection criteria

#### 2.1.1 Study type

Researchers will include all the RCTs of acupuncture at 3 anti-fatigue acupoints on CRF, without publication status. While others, such as non-RCTs, animal studies, case reports, will be excluded.

### 2.2 Participants

Regardless of types of cancer, gender, ages, nationalities, and educational status, the patients with CRF will be included. Firstly, the included participants should be diagnosed with cancer from cytological diagnosis, pathological diagnosis, or clinical diagnosis based on the past or current diagnosis standards or guidelines of cancer, such as New Standard for Diagnosis and Treatment of Malignant Tumors, which is formulated by China Cancer Association. Secondly, the included patients with cancer should conform to the definitions of CRF according to the diagnosis standards, such as International Classification of Diseases Tenth Edition.

### 2.3 Interventions

#### 2.3.1 Experimental interventions

The intervention group included will use acupuncture at 3 anti-fatigue acupoints based on routine regimens of the control group, or only acupuncture at 3 anti-fatigue acupoints, which includes electroacupuncture and ordinary acupuncture. Acupuncture at 3 anti-fatigue acupoints is different from traditional acupuncture. Acupuncture at 3 anti-fatigue acupoints refers to only inserting needles into 3 acupoints (Sishen Needle, Neiguan, and Zusanli) at a time, while traditional acupuncture means selecting classic acupoints of channels and collaterals to resist fatigue. The time and frequency of treatment will not be restricted. If the intervention group uses electrical or manipulation stimulation at 3 anti-fatigue acupoints, it will be excluded.

### 2.4 Comparator interventions

The patients in control group will receive several interventions comprised of no treatment, placebo acupuncture, sham acupuncture, drug or other treatments (such as aerobic exercise, dietotherapy, and health education).

Several comparisons will be analyzed as following:

(1) acupuncture at 3 anti-fatigue acupoints compared with no treatment;
(2) acupuncture at 3 anti-fatigue acupoints compared with placebo acupuncture or sham acupuncture;
(3) acupuncture at 3 anti-fatigue acupoints plus drug or other treatments compared with drug or other treatments;
(4) acupuncture at 3 anti-fatigue acupoints plus drug or other treatments compared with placebo acupuncture or sham acupuncture plus drug or other treatments.

### 2.5 Outcome measures

The primary outcome measures will be certain common scales which reflect the patients’ fatigue degree or life quality, such as piper fatigue scale (PFS), brief fatigue inventory, fatigue symptom inventory, Karnofsky performance status, The Europe Organization for Research and Treatment of Cancer, Quality of Life Questionnaire-Core 30, and so on.

The secondary outcome measures will be the blood index such as 5-hydroxytryptamine (5-HT), interleukin-6 (IL-6), tumor necrosis factor-α (TNF-α), and interferon.

### 2.6 Information sources

#### 2.6.1 Search strategy and identification of studies

Related literatures published from inception to October 2018, will be retrieved from the following databases: Cochrane Library, Embase, PubMed, 4 Chinese databases (Chinese National Knowledge Infrastructure, Chinese Science and Technology Periodicals Database, Chinese BioMedical Database and Wanfang Database), a Japanese database (CiNii) and a Korean database (OASIS). The search terms will be “cancer-related fatigue” OR “CRF” AND “acupuncture at three anti-fatigue acupoints” OR “Pi three-needle” OR “Pi 3-needle” OR “Pisan Needle” OR “Pisanzhen” OR “Jin's three-needle” OR “Jin's 3-needle” OR “3-points for fatigue” OR “moxibustion” OR “acupuncture” OR “electroacupuncture” OR “needle.” In Chinese, Korean, and Japanese databases, we will use the corresponding translations of these search terms to perform electronic searches. Detailed search strategy is shown as Table 1.

**Table 1:**
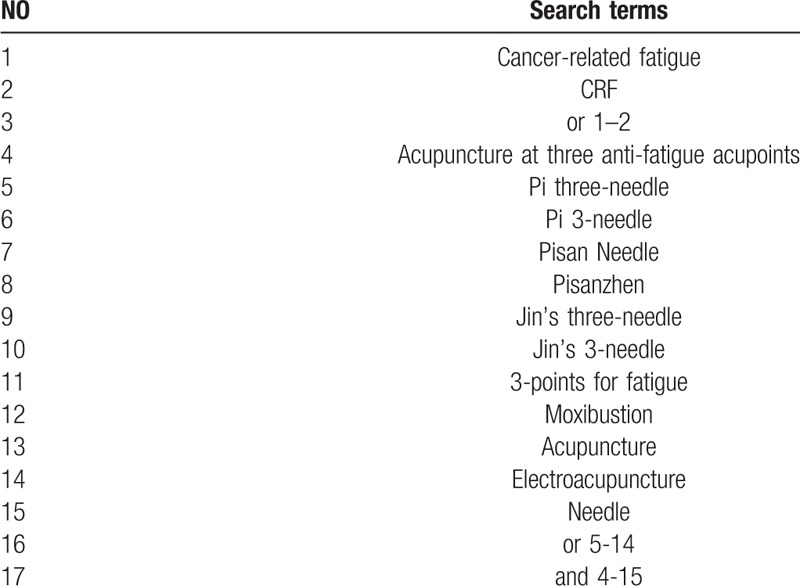
Search strategy used in for the PubMed database.

Other resources including references list of anteriorly published reviews and relevant conference proceedings will be searched for potential eligible studies.

**Figure 4:**
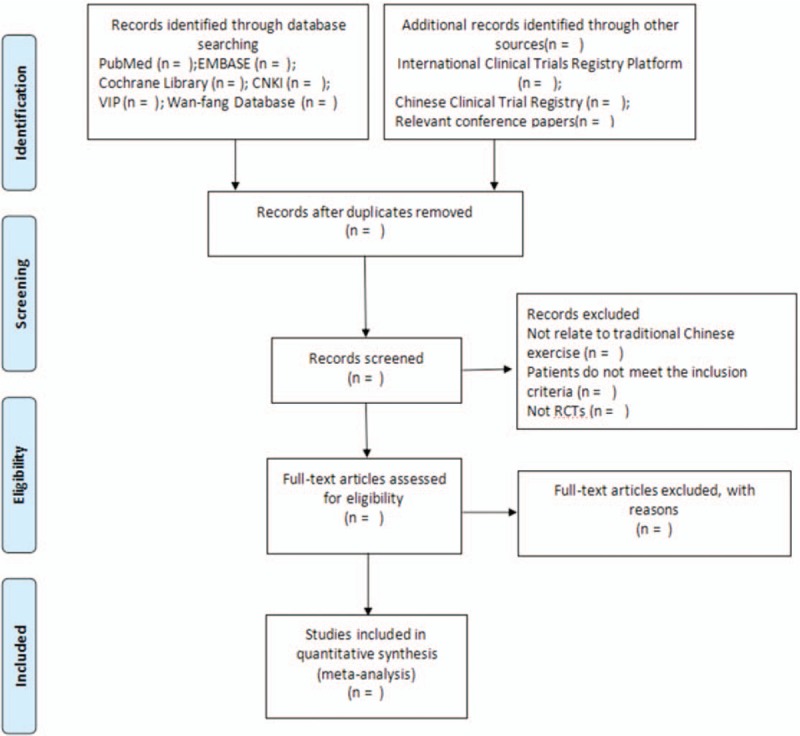
Flow diagram of the study selection process.

The reviewers (TL and YZX) will scan full articles independently. When divarication generates during the course and cannot be solved through discussion between 2 reviewers, the third reviewer (JY) will be consulted for arbitration. A Preferred Reporting Items for Systematic Reviews and Meta-Analyses flow chart will be drawn to describe the study selection process and explain the reason for inclusion and exclusion (http://www.prisma-statement.org) (Fig. 4).

### 2.7 Selection of studies

The articles with repeated results, unclear outcome data or unavailable related information, will be excluded. The reviewers (TL and YZX) will review titles and abstracts to select potential eligible literature and scan full articles to select the literature which can conform to the inclusion criteria, independently. A third reviewer (JY) will solve the disagreement between 2 reviewers. Endnote V.X7 will be used for literature managements and duplication removals.

### 2.8 Data extraction and management

The reviewers (TL and YZX) will independently extract data by using a data extraction sheet. The data will include the following items: general information (first author and publication year), study design, randomization methods, allocation concealment, blind method, inclusion and exclusion criteria of participants, participant's characteristics (sex, age, and duration of disease), sample size, interventions, primary and secondary outcomes, outcome assessment methods, side effects, and follow-up. Any disagreements will be discussed by the reviewers to reach consensus, and if necessary, it will be consulted with the third reviewer.

### 2.9 Addressing missing data

Researchers will contact with the original authors by telephone or email to request incomplete or missing data when necessary. If the information cannot be supplemented sufficiently in this way, researchers will abandon it. The potential impact of missing data will be considered in the discussion section.

### 2.10 Risk of bias in included studies

For assessing the risk of bias, researchers will utilize Cochrane collaboration's tool, which contains the following domains: generation of random sequence, allocation concealment, blinding method of participants, blinding method of outcome assessors, selective outcome reporting, incomplete result, and other bias. In order to describe the risk of bias in each study clearly, researchers will categorize the risk into unclear, low or high.

### 2.11 Statistical analysis

#### 2.11.1 Data synthesis and analysis

Stata V.13.0. software will be performed to analyze the data we obtained. We will calculate standard mean difference with 95% confidence intervals (CIs) for continuous outcomes and risk ratios with 95% CIs for dichotomous outcomes.

For assessing the heterogeneity, the *Q* and *I*^2^ test statistics will be performed. For the *Q* statistic, we will interpret the value of *P* < .1 as significant differences. For the *I*^2^ statistic, *I*^2^ > 50% indicates strong heterogeneity, *I*^2^ = 25% to 50% means moderate heterogeneity, and *I*^2^ < 25% represents no heterogeneity. If the data is considered as little heterogeneity, the fixed effect model will be adopted, or else the random effect model will be adopted.

### 2.12 Additional analyses

To explore the potential sources of heterogeneity, we will perform subgroup analysis, partial sequence analysis, and sensitivity analysis on the basis of various literature characteristics, such as locations of study, types of study, sample size, different participant conditions, different interventions and outcome measures, quality of study, and other related factors. We will use the extracted data to conduct quantitative analysis. But if the data is insufficient, qualitative analysis will replace it.

### 2.13 Assessment of reporting biases

When the included literatures are more than 10, we will use a funnel plot to assess the reporting bias. Egger and Begg tests will be utilized to evaluate funnel plot symmetry that *P* < .1 will be considered as showing statistical significance.

### 2.14 Quality of evidence

The quality of evidence of the included literatures will be judged by the grading of recommendations assessment, development, and evaluation. And the quality of evidence will be evaluated according to 4 levels: very low, low, moderate, or high.

### 2.15 Ethics and dissemination

Disseminating this systematic review and meta-analysis in a peer reviewed publication is our expected goal. The finding will furnish information about the safety and effect of acupuncture at 3 anti-fatigue acupoints on CRF in patients with cancer. As participants’ privacy will not be involved in this study, ethical approval is not required.

## 3 Discussion

According to TCM theories, acupuncture alone or in combination with moxibustion is often used to tonify qi and blood. To put acupuncture treatment into good use, it is of significance to select acupoints in treating CRF. Therefore, acupuncture at 3 anti-fatigue acupoints will be applied to the subjects in this study. Three anti-fatigue acupoints include Sishen Needle, PC6, ST36. Sishen Needle, is designated the sites 1.5 cun anterior, posterior left and right to Baihui (GV 20). It was suggested that Sishen Needle could regulate platelet 5-HT and plasma ACTH, which is helpful for the symptoms related to CRF.^[23]^ In addition, ST36 is able to Tonify qi and blood, relieve abdominal discomforts and digestive or elimination dysfunctions.^[24]^ Particularly, ST36 regulates immunity and reduces inflammation, which can improve the long term effects cause by related cancer treatments.^[25,26]^ Another recent study concluded that acupuncture at ST36 and PC6 significantly lowered the level of the data of heart rate and oxygen consumption, indicating a fatigue relief.^[27]^

Consequently, acupuncture at 3 anti-fatigue acupoints is theoretical in treating CRF. Nevertheless, the efficacy of acupuncture at 3 anti-fatigue acupoints compared with pharmacological interventions remains unknown. Additionally, there is insufficient evidence that acupuncture at 3 anti-fatigue acupoints is able to treat CRF. The aim of this review is to systematically assess the effect of acupuncture at 3 anti-fatigue acupoints in the treatment of CRF in patients with cancer. Researchers hope to use enough studies to make sure that adequate power for the meta-analysis. Also, researchers expect to find out whether acupuncture at 3 anti-fatigue acupoints has a positive effect on primary prevention of CRF in patients with cancer. Generally speaking, this review will be the first to evaluate the impact of acupuncture at 3 anti-fatigue acupoints on primary prevention of CRF in patients with cancer. The outcome of this review may be beneficial to establish a better approach to prevent CRF in patients with cancer and provide reliable evidence for its application.

## Author contributions

MXL, JY, YQY, and JTL conceived the study and drafted the protocol. YZX, TL, WJZ, LZL revised it. MXL, JY, YZX, and SLJ developed the search strategies, conducted data collection and analyzed independently. All authors have approved the final manuscript.

**Conceptualization:** Muxi Liao, Shuliang Ji.

**Data curation:** Yaqin Yang.

**Investigation:** Shuliang Ji.

**Methodology:** Wenjing Zhao.

**Project administration:** Tong Lin.

**Resources:** Jiao Yan.

**Software:** Zongyao Li.

**Writing – original draft:** Muxi Liao, Jiao Yan, Yizi Xie.

**Writing – review and editing:** Lizhu Lin, Jietao Lin.
